# Memory B Cells and Antibody Responses to Vaccines in Infected Children (MARVIC) With Recurrent Infections: Protocol for a Prospective Observational Study

**DOI:** 10.2196/101835

**Published:** 2026-10-13

**Authors:** Jalilah Jamaluddin, Siti Mardhiana Mohamad, Mohd Azri Zainal Abidin, Khairoon Nisa Mohamed Nashrudin, Malina Osman, Intan Hakimah Ismail

**Affiliations:** 1 Clinical Immunology Unit, Department of Paediatrics, Faculty of Medicine and Health Sciences, Universiti Putra Malaysia, Serdang, Selangor, 43400, Malaysia, 60 3-9769 2608; 2 Advanced Medical Research in Allergy and Clinical Immunology (AMRAC), Hospital Sultan Abdul Aziz Shah, Universiti Putra Malaysia, Serdang, Selangor, Malaysia; 3 Department of Community Health, Advanced Medical and Dental Institute, Universiti Sains Malaysia, Bertam, Penang, Malaysia; 4 Department of Community Health, Faculty of Medicine and Health Sciences, Universiti Putra Malaysia, Serdang, Selangor, Malaysia

**Keywords:** B-cell deficiency, humoral immunity, immunoglobulin G subclasses, IgG subclasses, immunoglobulin G subclass deficiency, IgG subclass deficiency, memory B cells, primary immunodeficiency, recurrent infections, reference range, specific antibody deficiency, vaccine-specific antibody responses

## Abstract

**Background:**

Recurrent infections are a common concern in pediatric practice and may prompt evaluation for an underlying inborn error of immunity, particularly when infections are severe, persistent, unusual, or recurrent. Recurrent sinopulmonary infections such as pneumonia, sinusitis, and otitis media may indicate a humoral immune defect, as antibody deficiencies are among the most common inborn errors of immunity. Laboratory tests typically include measurement of serum immunoglobulins (immunoglobulin G [IgG], immunoglobulin A [IgA], and immunoglobulin M [IgM]) and IgG subclasses (IgG1, IgG2, IgG3, and IgG4), assessment of vaccine-specific antibody responses, and enumeration of B-cell subsets. Together, these tests assess the quantitative and functional components of humoral immunity. However, pediatric reference intervals for these immunological parameters have not been established in Malaysia.

**Objective:**

This study aims to characterize humoral immune parameters among Malaysian children with recurrent infections, compare these parameters with those of healthy children, and establish age-specific reference values in a healthy pediatric population.

**Methods:**

The MARVIC (Memory B Cells and Antibody Responses to Vaccines in Infected Children) study is a prospective observational comparative study incorporating a vaccine challenge follow-up in children with recurrent infections and age-matched healthy controls. Eligible participants will undergo baseline assessment of serum immunoglobulins and IgG subclass concentrations using nephelometry, B-cell subsets using flow cytometry, and vaccine-specific antibody responses using enzyme-linked immunosorbent assays. They will then receive the tetanus toxoid and 23-valent pneumococcal polysaccharide vaccines, followed by repeat blood sampling 28 to 30 days later to evaluate functional antibody responses. The co–primary outcomes are between-group differences in IgG subclass concentrations, vaccine-specific antibody responses, and B-cell subsets between children with recurrent infections and healthy controls. The secondary outcome is the establishment of age-specific reference intervals for these immunological parameters among healthy children.

**Results:**

This study was funded in December 2024, and participant recruitment and data collection are ongoing. As of May 2026, a total of 145 participants had been recruited, comprising 60 (41.4%) children with recurrent infections and 85 (58.6%) healthy controls, all of whom had completed postvaccination follow-up sampling. Data collection and analysis are expected to be completed by December 2026, with publication of the findings anticipated in May 2027.

**Conclusions:**

This study will provide a comprehensive evaluation of the humoral immune profile of Malaysian children with recurrent infections. The findings may improve the identification of clinically relevant antibody deficiencies and support the interpretation of pediatric immunological investigations in Malaysia.

## Introduction

### Background

Recurrent infections in children are a common concern in pediatric and primary care settings. Although many affected children are otherwise healthy, recurrent, severe, persistent, or unusual infections may indicate an underlying inborn error of immunity (IEI), previously termed a primary immunodeficiency disease. IEIs are a heterogeneous group of disorders caused by defects in immune system development and/or function, leading to increased susceptibility to infections and immune dysregulation [1]. These disorders are classified according to the immune component or pathway affected [2]. Antibody deficiencies are among the most frequently diagnosed IEIs and commonly present via recurrent sinopulmonary infections, such as sinusitis, otitis media, bronchitis, and pneumonia [3,4].

The human immune system comprises 5 immunoglobulin classes: immunoglobulin G (IgG), immunoglobulin A (IgA), immunoglobulin M (IgM), immunoglobulin D (IgD), and immunoglobulin E (IgE) [5]. IgG is the most abundant immunoglobulin in plasma and plays a crucial role in long-term protection against pathogens. IgG is divided into four subclasses—IgG1, IgG2, IgG3, and IgG4—each of which differs in antigen specificity and effector function [6]. IgG1 and IgG3 respond predominantly to protein antigens and effectively promote complement activation and Fc receptor–mediated phagocytosis. IgG2 is particularly important in responses to carbohydrate antigens, including bacterial capsular polysaccharides, but activates complements less efficiently. IgG4 has minimal capacity to activate the classic complement pathway and is generally associated with inflammatory effector activity [6].

IgG subclass deficiencies (IGGSDs) are characterized by persistently low levels of one or more IgG subclasses despite a normal total IgG level. Its clinical significance should be assessed in conjunction with the patient’s infection history and functional antibody responses, as isolated reductions may be transient or clinically insignificant, particularly in young children. For example, IgG1 deficiency may result in hypogammaglobulinemia because IgG1 constitutes 60% to 70% of total IgG. IgG2 and IgG3 deficiencies are reported most frequently, whereas IgG2 deficiency may also occur in association with other IEIs, including IgA deficiency and ataxia-telangiectasia [5,7-10].

Specific antibody deficiency (SAD) is characterized by an impaired antibody response to polysaccharides despite normal total immunoglobulins and IgG subclass levels. It is usually identified through measurement of serotype-specific antibody responses before and after pneumococcal polysaccharide vaccination [11,12]. However, SAD should generally not be diagnosed in children younger than 2 years because responses to unconjugated polysaccharide antigens may be physiologically immature.

Children with IGGSD and SAD may experience recurrent respiratory tract infections, particularly those caused by encapsulated bacteria such as *Streptococcus pneumoniae* and *Haemophilus influenzae*. The clinical significance of IGGSD and SAD is difficult to determine in young children because immunoglobulin concentrations and antibody responses change with age [11,13,14]. Accurate evaluation is therefore important to avoid both delayed diagnosis and overdiagnosis. Management may include prompt treatment of infections; prophylactic antibiotics; and, in selected patients, immunoglobulin replacement therapy [15].

Evaluation of humoral immunity in children with recurrent infections typically involves measurement of serum immunoglobulins and IgG subclass concentrations as well as antibody responses to protein and polysaccharide vaccine antigens [16-18]. Vaccine challenge testing provides a functional assessment of antibody production, and it complements quantitative measurements [17-21]. Tetanus toxoid is a protein antigen that induces a T-cell–dependent antibody response. In contrast, the 23-valent pneumococcal polysaccharide (Pneumovax 23) vaccine contains unconjugated capsular polysaccharide antigens and predominantly evaluates T-cell–independent antibody responses. Assessment of responses to both antigen types may help identify subtle abnormalities of antibody production in children with recurrent infections.

Memory B cells contribute to long-term humoral immunity by mounting rapid antibody responses after re-exposure to an antigen [22-24]. Phenotypic B-cell subsets include switched memory B cells (CD19^+^CD27^+^IgM⁻IgD⁻), unswitched or IgM memory B cells (CD19^+^CD27^+^IgM^+^IgD^+^), IgM-only memory B cells (CD19^+^CD27^+^IgM^+^IgD⁻), and IgD-only memory B cells (CD19^+^CD27^+^IgM⁻IgD^+^) [25]. These populations arise through T-cell–dependent and T-cell–independent pathways. Reduced memory B-cell populations may impair recall responses and increase susceptibility to recurrent infections [26]. Therefore, evaluating the memory B-cell compartment may provide additional information in children with suspected humoral IEIs.

To the best of our knowledge, this is the first study in Malaysia to evaluate IgG subclasses, vaccine-specific antibody responses, and memory B-cell populations concurrently in children with recurrent infections and healthy controls. This protocol describes the rationale, design, laboratory methods, and planned analyses of the MARVIC (Memory B Cells and Antibody Responses to Vaccines in Infected Children) study.

### Study Rationale

Children with recurrent infections often pose a diagnostic challenge, particularly in settings where access to specialized immunological tests is limited. Concurrent assessment of IgG subclasses, vaccine-specific antibody responses, and memory B-cell subsets may identify subtle humoral immune abnormalities that are not detected by total immunoglobulin measurements alone. Comparison with locally recruited healthy children will also provide preliminary pediatric reference data relevant to the Malaysian population. These findings may support earlier recognition of clinically significant humoral immune dysfunction and facilitate more appropriate clinical management.

### Study Aim and Objectives

The purpose of the MARVIC study is to characterize humoral immunity in children with recurrent infections and compare the findings with those in healthy children. The study will assess serum immunoglobulin and IgG subclass concentrations; pre- and postvaccination antibody concentrations; and B-cell subsets, including memory B-cell populations. It will also examine associations between these immunological parameters and relevant clinical characteristics and establish preliminary age-specific reference intervals among healthy Malaysian children.

The specific objectives are as follows:

- To describe the demographic and clinical characteristics of children with recurrent infections
- To compare serum immunoglobulins and IgG subclass concentrations between children with recurrent infections and healthy controls
- To compare B-cell subsets, including memory B-cell populations, between children with recurrent infections and healthy controls
- To compare antibody responses to pneumococcal polysaccharide and tetanus toxoid vaccines between children with recurrent infections and healthy controls
- To examine associations between immunological parameters and clinical characteristics among children with recurrent infections
- To establish preliminary age-specific reference intervals for IgG subclasses, B-cell subsets, and vaccine-specific antibody responses in healthy Malaysian children

## Methods

### Study Design and Setting

The MARVIC study is a prospective observational comparative study incorporating a diagnostic vaccine challenge follow-up. It will evaluate serum immunoglobulins, IgG subclasses, pre- and postvaccination antigen-specific antibody concentrations, and memory B-cell populations in children with recurrent infections and healthy controls. Participants will be recruited from 2 tertiary hospitals: Sultan Abdul Aziz Shah Hospital, Universiti Putra Malaysia, and Sultan Idris Shah Hospital, Serdang. All laboratory procedures will be conducted at the Clinical Immunology Laboratory, Faculty of Medicine and Health Sciences, Universiti Putra Malaysia. Pediatricians of both hospitals will serve as site coinvestigators.

### Participant Eligibility

Potential participants will be identified and screened at the pediatric clinics and wards of both study sites. Children admitted with an acute infection will be assessed for enrollment only after clinical recovery. Following preliminary screening, potential participants will undergo baseline immunological assessment. Final eligibility will be confirmed after serum IgG, IgA, and IgM concentrations and T-, B-, and natural killer–cell counts are shown to be within age-specific reference intervals. The eligibility criteria for children with recurrent infections and healthy controls are summarized in Textboxes 1 and 2, respectively.

Textbox 1. **Eligibility criteria for children with recurrent infections.**

**Inclusion criteria**

- Age of 2-18 years
- History of recurrent infections, defined for this study as 2 or more clinically documented infections within the preceding 12 months
- Clinically well and free from acute infection at enrollment
- Baseline serum immunoglobulin G, immunoglobulin A, and immunoglobulin M concentrations within age-specific reference intervals
- Baseline T-, B-, and natural killer–cell counts within age-specific reference intervals
- No previous receipt of the 23-valent pneumococcal polysaccharide vaccine
- Ability to return for postvaccination blood sampling 28-30 days after vaccination
- Written parental or guardian consent and child assent when applicable

**Exclusion criteria**

- Known or previously diagnosed inborn error of immunity or other immune disorders
- Chronic disease that may independently affect immune parameters, including malignancy
- Receipt of intravenous or subcutaneous immunoglobulin therapy within the preceding 6 months
- Current treatment with immunotherapy or immunosuppressive or immunomodulatory medication
- Syndromic condition known or suspected to affect immune function
- History of severe allergic reaction to a component of either study vaccine or another contraindication to vaccination

Textbox 2. **Eligibility criteria for healthy controls.**

**Inclusion criteria**

- Age of 2-18 years
- Clinically healthy and free from acute infection or other acute illness at enrollment
- Baseline serum immunoglobulin G, immunoglobulin A, and immunoglobulin M concentrations within age-specific reference intervals
- Baseline T-, B-, and natural killer–cell counts within age-specific reference intervals
- No previous receipt of the 23-valent pneumococcal polysaccharide vaccine
- Ability to return for postvaccination blood sampling 28-30 days after vaccination
- Written parental or guardian consent and child assent when applicable

**Exclusion criteria**

- History of recurrent infections as defined for the recurrent infection group
- Known or previously diagnosed inborn error of immunity or other immune disorder
- Chronic disease that may independently affect immune parameters, including malignancy
- Receipt of intravenous or subcutaneous immunoglobulin therapy within the preceding 6 months
- Current treatment with immunotherapy or immunosuppressive or immunomodulatory medication
- Syndromic condition known or suspected to affect immune function
- History of severe allergic reaction to a component of either study vaccine or another contraindication to vaccination

### Sample Size Calculation

The sample size was based on the comparison of the proportion of participants with IGGSD between children with recurrent infections and healthy controls. On the basis of previous studies, the expected proportions were 78.7% in children with recurrent infections and 50% in healthy controls [27,28]. Assuming equal group allocation, 80% power, and a 2-sided α level of .05, a minimum of 42 participants per group was required. After allowing for 10% attrition, the adjusted sample size was 47 participants per group. A conservative recruitment target of 55 participants per group was adopted, yielding a total target sample size of 110 participants. The calculation was performed using G*Power (version 3.1.9.7).

### Data Collection

Demographic data will include date of birth, age, gender, ethnicity, dates of clinic visits, dates of hospital admission and discharge, and duration of hospitalization. Clinical data will include immunization and feeding histories, parental consanguinity, personal and family histories of allergic disease, and family history of recurrent infections. Relevant clinical manifestations will include lymphadenopathy or lymphadenitis, finger clubbing, hepatomegaly, splenomegaly, oral ulcers, oral candidiasis, skin and perianal infections, otitis media, sinusitis, conjunctivitis, pneumonia, bronchiectasis, chronic lung disease, pleural effusion, vomiting, diarrhea, osteomyelitis, arthritis, parasitic infections, global developmental delay, and failure to thrive. Infection-related data will include age at onset, frequency and types of infections, causative organisms, infection severity, and number of hospitalizations. Available laboratory results, including complete blood counts, complement C3 and C4 concentrations, isohemagglutinin titres, lymphocyte subset enumeration, and total IgE concentrations will be retrieved from the medical records. Serum immunoglobulins and IgG subclasses, vaccine-specific antibody concentrations, and B-cell subsets will be measured as part of the study.

### Blood and Serum Collection

At enrollment, baseline blood samples will be collected before vaccination. For each participant, 3 to 5 mL of blood will be collected into each of 2 tubes: an ethylenediaminetetraacetic acid (EDTA) tube and a plain, nonheparinized tube. Following blood collection, participants will receive the Pneumovax 23 and tetanus toxoid vaccines.

The EDTA-anticoagulated blood will be processed immediately for B-cell subset analysis. Blood collected in the plain tube will be centrifuged at 3000 rpm for 5 minutes. At least 1 mL of the resulting serum will be divided into 2 Eppendorf tubes: one for measurement of immunoglobulins and IgG subclasses via nephelometry and the other for measurement of baseline vaccine-specific antibody concentrations via enzyme immunoassay. All specimens will be labeled with a unique coded study identifier.

At 28 to 30 days after vaccination, a second blood sample will be collected in a plain tube for postvaccination antibody concentrations. The serum will be separated and aliquoted for enzyme immunoassay. All serum samples will be stored at –20 °C in the Clinical Immunology Laboratory until analysis. Paired pre- and postvaccination serum samples will be analyzed in the same assay run whenever feasible to minimize interassay variation. Residual samples will be discarded after completion of all planned analyses in accordance with the approved study protocol.

### Measurement of Immunoglobulins and IgG Subclasses via Nephelometry

Serum IgG, IgA, IgM, and IgG subclass concentrations will be measured using the BN ProSpec nephelometric analyzer (Siemens Healthineers) in accordance with the manufacturer’s instructions. Reagents, calibrators, and quality control materials will be loaded onto the analyzer. A calibration curve will be generated using automated serial dilutions of the appropriate calibrators. Following successful calibration and verification of quality control results, serum samples will be analyzed using the instrument’s automated sampling, dilution, and measurement procedures. Results will be reported in grams per liter.

### Measurement of Antipneumococcal and Antitetanus IgG via Enzyme-Linked Immunosorbent Assay

Antitetanus toxoid IgG and anti–pneumococcal capsular polysaccharide (PCP) IgG concentrations will be measured using VaccZyme antitetanus toxoid IgG and anti–anti-PCP IgG enzyme-linked immunosorbent assay kits (The Binding Site) in accordance with the manufacturer’s instructions. The anti-PCP IgG assay uses microwells coated with a mixture of capsular polysaccharide antigens representing the 23 pneumococcal serotypes included in Pneumovax 23: 1, 2, 3, 4, 5, 6B, 7F, 8, 9N, 9V, 10A, 11A, 12F, 14, 15B, 17F, 18C, 19F, 19A, 20, 22F, 23F, and 33F. The antitetanus IgG assay uses microwells coated with tetanus toxoid antigens. For each assay, 100 μL of calibrator, control, and diluted (1:100) serum sample will be added to the designated microwells and incubated at room temperature for 30 minutes. After washing, 100 μL of conjugate solution will be added, followed by incubation at room temperature for 30 minutes. The wells will then be washed before the addition of 100 μL of 3,3’,5,5’-tetramethylbenzidine substrate. After incubation for 30 minutes at room temperature in the dark, the reaction will be stopped by adding 100 μL of stop solution containing 3M phosphoric acid. Optical density will be measured at 450 nm using a microplate reader within 30 minutes of stopping the reaction. Paired pre- and postvaccination serum samples from each participant will be analyzed in the same assay run whenever feasible to minimize interassay variation.

### B-Cell Subset Immunophenotyping via Flow Cytometry

B-cell subsets will be characterized using a BD FACSLyric (BD Biosciences) flow cytometer, and data will be analyzed using the BD FACSuite software (version 1.5; BD Biosciences). The 6-color antibody panel will comprise CD19-PerCP-CY5.5, CD24-APC-H7, CD27 APC, CD38 PE-Cy7, IgD PE, and IgM BB515. Before sample acquisition, instrument performance and fluorescence compensation will be verified in accordance with the laboratory’s standard operating procedures. The BD FACS lysing solution (BD Biosciences) will be prepared as a 1X working solution by diluting the 10X concentrate with deionized water at a ratio of 1:10. Briefly, 100 µL of EDTA-anticoagulated whole blood will be mixed with approximately 5 mL of stain buffer in a 12 × 75 mm tube and centrifuged at 300 × *g* for 5 minutes. After removal of the supernatant, the appropriate volume of fluorochrome-conjugated monoclonal antibody cocktail will be added to the cell pellet. The tubes will be gently vortexed and incubated for 15 minutes at room temperature in the dark. Subsequently, 2 mL of 1X BD FACS lysing solution will be added, and the tube will be gently vortexed and incubated for 10 minutes at room temperature in the dark. The cells will then be centrifuged at 300 × *g* for 5 minutes, and the supernatant will be discarded. The cells will be washed twice with 2 to 3 mL of stain buffer and centrifuged at 300 × *g* for 5 minutes after each wash. Following the final wash, the cell pellet will be resuspended in 500 µL of stain buffer and acquired on the flow cytometer. B cells will be identified based on CD19 expression and classified into phenotypic subsets according to the expression of CD24, CD27, CD38, IgD, and IgM. The flow cytometry gating strategy for B-cell subsets will be adapted from a previously published protocol [29].

### Interpretation of Immunological Findings

Serum IgG, IgA, and IgM concentrations will be interpreted using applicable age-specific reference intervals. IgG subclass concentrations will also be interpreted according to age-specific reference intervals, and values below the lower reference limit will be classified as reduced. B-cell subset percentages and absolute counts will be classified as below, within, or above the applicable age-specific reference intervals.

Vaccine-specific antibody responses will be evaluated using paired pre- and postvaccination antibody concentrations. Postvaccination anti-PCP IgG and antitetanus toxoid IgG concentrations will be interpreted using the predefined assay-specific thresholds of 110 mg/L and 0.15 IU/mL, respectively [20,30]. The magnitude of response will also be evaluated by comparing postvaccination concentrations with baseline concentrations.

Participants with normal baseline total immunoglobulin concentrations and major lymphocyte counts but one or more abnormalities in IgG subclass concentrations, vaccine-specific antibody responses, or B-cell subsets will be classified as having an immunological abnormality for the purposes of the study. These study classifications will not, by themselves, be considered definitive clinical diagnoses of an IEI.

### Study Outcomes and Analytical Hierarchy

The co–primary outcomes are between-group differences in IgG subclass concentrations, vaccine-specific antibody responses, and B-cell subset percentages and absolute counts between children with recurrent infections and healthy controls. Vaccine-specific antibody responses will be assessed by comparing pre- and postvaccination antibody concentrations within each group and comparing the change in antibody concentrations and the proportion of participants achieving an adequate response between the 2 groups. The secondary outcome is the establishment of preliminary age-specific reference intervals for the immunological parameters among healthy controls. Exploratory analyses will examine associations between immunological parameters and relevant demographic and clinical characteristics.

### Establishment of Reference Intervals

Preliminary age-specific reference intervals will be established using data from eligible healthy controls who satisfy all criteria presented in Textbox 2. Reference intervals will be determined for serum immunoglobulin and IgG subclass concentrations, B-cell subsets, and vaccine-specific antibody concentrations. The distribution and skewness of the data will first be assessed. Potential univariate outliers for each immunological parameter will be identified using the method by Tukey. Mahalanobis distance will be applied as an additional method to identify potential multivariate outliers while accounting for covariance among the immunological parameters. Observations identified as potential outliers will be reviewed individually and excluded only when supported by a clinically or analytically justifiable reason. Reference intervals will be estimated in accordance with the Clinical and Laboratory Standards Institute C28-A3 guideline. Because fewer than 120 healthy participants are expected within each age partition, the robust method will be used provided that the minimum sample size requirements are satisfied. The lower and upper reference limits will encompass the central 95% of the reference distribution, corresponding to the 2.5th and 97.5th percentiles, respectively. Given the anticipated sample size within each age group, the findings will be interpreted as preliminary age-specific reference intervals. Reference interval analyses will be performed using the MedCalc statistical software (version 23.0.8; MedCalc Software Ltd).

### Age Stratification

Humoral immune parameters vary throughout childhood because of progressive immune system development and maturation. Participants will therefore be stratified into 3 prespecified age groups: 2 to 4 years, 5 to 9 years, and 10 to 18 years. These age partitions were prespecified based on established pediatric immunology practice and published age-specific reference intervals [31,32]. Age-stratified analyses will be conducted for serum immunoglobulins, IgG subclasses, B-cell subsets, and vaccine-specific antibody responses.

### Data Management, Confidentiality, Quality Control, and Missing Data

All study-related information will be managed confidentially in accordance with applicable laws and regulations. Each participant and corresponding biological specimens will be assigned a unique coded study identifier. The linkage file connecting the study identifier with participants’ personal information will be stored separately from the research dataset and will be accessible only to authorized study personnel. Signed informed consent forms and other paper records containing personal identifiers will be stored in locked cabinets. Electronic research data will be maintained in password-protected files, with access restricted to authorized members of the research team. Participants’ identities will not be disclosed in publications or presentations arising from the study.

Laboratory analyses will be performed using standard operating procedures, calibrated instruments, and appropriate quality control materials. Pre- and postvaccination serum samples from the same participant will be analyzed in the same assay run to minimize interassay variation. Laboratory results and data entries will be reviewed for completeness and accuracy before statistical analysis.

Serum immunoglobulins, IgG subclasses, and B-cell subsets will be evaluated using baseline samples. Vaccine response analyses will require paired pre- and postvaccination samples collected 28 to 30 days apart. The number and proportion of participants with missing data will be reported for each outcome together with the reasons for missingness where available. Participants who do not complete postvaccination blood sampling will be excluded only from the paired vaccine response analyses; their available baseline data may still be included in other relevant analyses.

### Statistical Analysis

Statistical analysis will be conducted using SPSS Statistics for Windows (version 27.0; IBM Corp). Continuous variables will be presented as means and SDs when approximately normally distributed and as medians with IQRs when skewed. The distribution of continuous variables will be assessed using graphical methods and appropriate normality tests. Categorical variables will be presented as frequencies and percentages. Skewed immunological measurements will be transformed where appropriate before inferential analysis. Comparisons of continuous variables between 2 independent groups will be performed using the independent-sample *t* test (2-tailed) or Mann-Whitney *U* test, as appropriate. Comparisons involving more than 2 groups will be performed using one-way ANOVA or the Kruskal-Wallis test. Pre- and postvaccination antibody concentrations within each group will be compared using the paired-sample *t* test or Wilcoxon signed-rank test. Categorical variables will be compared using the chi-square test or Fisher exact test, as appropriate. Effect estimates, 95% CIs, and exact *P* values will be reported. All statistical tests will be 2 sided, and *P*<.05 will be considered statistically significant unless adjustment for multiple comparisons is required. Where multiple comparisons are performed within a group of related immunological outcomes, the significance threshold will be adjusted using the Bonferroni correction to reduce the risk of type I error.

### Adjustment for Potential Confounders

Potential confounders will be identified a priori based on biological plausibility, clinical relevance, and evidence from previous studies. Age, sex, ethnicity, relevant vaccination history, and other relevant clinical characteristics will be considered. The covariates included in each adjusted analysis will be selected according to the specific relationship between study group and immunological outcome rather than entered automatically into every model. Multivariable linear regression will be used for continuous outcomes, whereas binary logistic regression will be used for dichotomous outcomes where appropriate. The number of covariates included in each model will be limited according to the available sample size to reduce the risk of overfitting. Unadjusted and adjusted effect estimates will be reported with 95% CIs.

### Ethical Considerations

This study protocol was reviewed and approved by the Medical Research and Ethics Committee, Ministry of Health Malaysia (NMRR-19-2299-49077), and the Ethics Committee for Research Involving Human Subjects, Universiti Putra Malaysia (JKEUPM-2023-337). Written informed consent will be obtained from the parent or legal guardian of each participant before any study-related procedure is performed. Assent will also be obtained from participating children where appropriate according to their age and level of understanding.

## Results

The study was funded by Universiti Putra Malaysia under the Geran Inisiatif Putra Siswazah grant scheme in December 2024. Participant recruitment and data collection are ongoing. As of May 2026, a total of 145 participants had been recruited, comprising 60 (41.4%) children with recurrent infections and 85 (58.6%) healthy controls, all of whom had completed postvaccination follow-up sampling. Data collection and analysis are expected to be completed by December 2026, and publication of the findings is anticipated in May 2027. We expect the study to provide a comprehensive profile of humoral immunological markers in children with recurrent infections. In addition, this study is expected to identify potential differences in IgG subclass levels, memory B-cell profiles, and vaccine-specific antibody responses between affected children and healthy controls and establish reference ranges for IgG subclasses, memory B-cell populations, and specific antibody responses in the Malaysian pediatric population.

## Discussion

### Expected Findings

The MARVIC study is expected to provide a comprehensive evaluation of humoral immune parameters in Malaysian children with recurrent infections who have normal total immunoglobulin concentrations and major lymphocyte counts. The combined assessment of IgG subclasses, vaccine-specific antibody responses, and B-cell subsets may identify subtle immunological abnormalities that are not detected through routine immunological screening.

The diagnostic vaccine challenge is an important component of the study because it allows for functional assessment of antibody production. The Pneumovax 23 vaccine primarily evaluates responses to polysaccharide antigens, whereas the tetanus toxoid vaccine evaluates responses to a protein antigen. Evaluating responses to these distinct antigen types may provide complementary information about humoral immune function. This is particularly relevant for children with recurrent sinopulmonary infections in whom IGGSD or impaired specific antibody production is suspected despite normal total immunoglobulin concentrations.

B-cell subset immunophenotyping may provide additional information about B-cell maturation and immunological memory. Differences in memory B-cell populations between children with recurrent infections and healthy controls may help clarify whether recurrent infections are associated with abnormalities that are not apparent from total B-cell counts alone. However, any abnormal B-cell subset measurement will need to be interpreted in relation to age, clinical presentation, and the other immunological findings rather than used independently to classify a participant as immunodeficient.

The study will also generate preliminary age-specific reference intervals for selected immunological parameters among healthy Malaysian children. Because immune parameters change during childhood, locally derived age-specific values may improve the interpretation of pediatric immunological investigations. These intervals will nevertheless be considered preliminary because the number of healthy participants within each age partition may be below that recommended for establishing definitive reference intervals.

Several limitations should be considered. The observational study design will identify associations but will not establish causality. The eligibility criteria restrict the population to children with normal serum IgG, IgA, and IgM concentrations and normal major lymphocyte counts; therefore, the findings may not be generalizable to children with broader or more severe immune deficiencies. In addition, the pooled pneumococcal antibody assay measures aggregate IgG against multiple PCPs and does not provide serotype-specific responses. Consequently, the findings from this assay should be interpreted as an assessment of overall vaccine-specific antibody response rather than as a definitive diagnosis of SAD. Recruitment from two study sites and potentially small numbers within individual age groups may further limit the generalizability and precision of the reference intervals.

Despite these limitations, the MARVIC study may improve understanding of humoral immune profiles in Malaysian children with recurrent infections and support the interpretation of IgG subclass, vaccine-specific antibody, and B-cell subset measurements. The findings may also provide a foundation for larger multicenter studies using serotype-specific pneumococcal antibody assays and larger healthy reference populations.

### Conclusions

The MARVIC study will provide an integrated evaluation of IgG subclasses, vaccine-specific antibody responses, and B-cell subsets in Malaysian children with recurrent infections and healthy controls. The findings may improve understanding of subtle humoral immune abnormalities in children with recurrent infections and support the interpretation of pediatric immunological investigations. The preliminary age-specific reference intervals generated among healthy children may also provide a foundation for larger multicenter studies aimed at establishing definitive reference intervals for the Malaysian pediatric population.

## Supplementary material

10.2196/101835 Peer Review Report 1 Peer review report from the Geran Inisiatif Putra Siswazah Grant Committee, Universiti Putra Malaysia.

## Abbreviations

EDTA: ethylenediaminetetraacetic acid
IEI: inborn error of immunity
IgA: immunoglobulin A
IgD: immunoglobulin D
IgE: immunoglobulin E
IgG: immunoglobulin G
IGGSD: immunoglobulin G subclass deficiency
IgM: immunoglobulin M
MARVIC: Memory B Cells and Antibody Responses to Vaccines in Infected Children
PCP: pneumococcal capsular polysaccharide
SAD: specific antibody deficiency
