# Supplementary material for: Memory B Cells and Antibody Responses to Vaccines in Infected Children (MARVIC) With Recurrent Infections: Protocol for a Prospective Observational Study
Source: JMIR Res Protoc. 2026 Oct 13;15:e101835. doi: 10.2196/101835 (PMC13660582; doi:10.2196/101835)
Supplement: Supplementary file 1 [file resprot-v15-e101835-s001.pdf]

UPM UNIVERSITI PUTRA MALAYSIA BERILMU BERBAKTI	**PERKHIDMATAN UTAMA PENYELIDIKAN DAN INOVASI**<br><br>**PEJABAT TIMBALAN NAIB CANSELOR (PENYELIDIKAN & INOVASI)**<br>**Kod Dokumen: PU/PY/BR04/NILAIGERAN**
	**BORANG PENILAIAN PERMOHONAN GERAN PENYELIDIKAN**<br>***EVALUATION FORM FOR RESEARCH GRANT APPLICATION***

MAKLUMAT PROJEK *PROJECT INFORMATION*

**Tajuk Projek** *Project Title*	Characterisation of Immunoglobulins (IqG, IgA, IgM), IgG Subclasses, and Specific Antibody Resposes AMong Healthy Malaysian Children
**Ketua Projek** *Project Leader*	Intan Hakimah Binti Ismail
**PTJ** *Faculty / Institute*	FPSK
**Jenis Geran** *Type of Grant*	GP-IPS
**Kluster / Bidang** *Cluster / Research Area*	HWB

Kriteria Penilaian *Assessment criteria*	Wajaran Markah *Weightage of marks*	Tahap *Level* *Please click cell to select level*	Keterangan *Indicator*	Markah *Marks*	Skor *Score*	Ulasan Panel *Panel Remarks*
**1.0 Title and first impression** (Title should reflect the content of the proposal, concise and instantly impressionable)	5%	Average	The title describes the content of the proposal, and only left brief positive impression on the reviewer	3	3.00	Participants will be from HPUPM and Hospital Serdang, suffice to represent Malaysian children?
**2.0 Executive summary** (Should include introduction, problem statement, justification, objectives of the study. Expected key findings, potential beneficiaries and key contribution of the proposed study should be described.)	5%	Average	The executive summary describe the content of the proposal rather accurately, and provide limited amount of additional informtion for the reviewer to quickly understand the study	3	3.00	To further justify the importance of characterisation of immunoglobulins and specific antibody responses in healthy Malaysian children.
**3.0 Scientific Merit (Described according to SMART criterion - Scientific, Measurable, Achievable, Realistic and within Time-frame)**						
**3.1 Important, relevant and current**	10%	Good	The proposed study is important, relevant and current to contribute the field of interest. Findings would attract significant interest from peers in the fields of expertise	4	8.00	
**3.2 Problem Statement & research question(s)**	10%	Average	Problem statement and research questions are present & organised, but not adequately elaborated	3	6.00	To elaborate on specific antibody responses in Literature Review
**3.3 Proposal objectives**	10%	Good	Objectives address the problem statement & research question, as well as all of the intent in the proposed study.	4	8.00	
**3.4 Scientific merit**	5%	Good	The proposal has very good scientific merit that justifies scientific research	4	4.00	
**3.5 Ethical issues** (Should not contain elements of misinformation, or touches sensitivities that could potentially mislead, deceive and contravene the law and moral norms of the society. Plagiarism or unethical/unauthorised use of proposals is strictly prohibited. Studies should not contravene prevailing laws and conventions on human and animal research, such as guaranteed anonymity, confidentiality and prevention against the abuse and exploitation of study subjects). *Significant ethical issues will disqualify the proposal from the reviewing process	10%	Average	The proposal has minor ethical issue, and can be considered for funding	3	6.00	To clarify if the study has started since ethics has been obtained (methodology) and planned to be renewed (milestones).

**4.0 Methodology (Clear and detailed description of methods. May consist of field work, sampling techniques, interview session, analysis ; lab work of different phases, experimental protocol, statistical analysis etc.)**						
**4.1 Methodology description**	**5%**	**Below Average**	Methodology is included but very disorganised, and not really aligned with the objectives	**2**	**2.00**	To clarify recruitment of subjects, they attending Paediatric clinic/admitted to ward but supposedly healthy children?<br>Inconsistency of location - HPUPM and Hospital Serdang (study design) but only Faculty of Medicine and Health Sciences metioned in study location.<br>Sample size - "in this study, a non-parametric approach was adopted" - has completed the study?<br>120 subjects - how about the distribution according to the different age groups?<br>CLSI refered to is of the year 2000, no updated version?
**4.2 Methodology attributes** (Able to achieve research objectives with minimal possibilities of extension & project delay)	**5%**	**Good**	Methods described are established and suited to the research objectives of the current study, with none or very little technical issues, and risk of delays	**4**	**4.00**	
**4.3 Supplementary materials for methodology included** (proposal may require research design & framework diagram, flow chart, Gantt chart, activities and milestones etc, as per required by the sponsor)<br><br>*Ignore this category if it is not required by the sponsor*	**5%**	**Good**	Supplementary materials included are complete, fully aligned with the methodology description and helped to explain the proposed study	**4**	**4.00**	No Gantt chart
**5.0 Project Impact (Describe as the magnitude of impact in terms of reach, depth and universality of the impact)**						
**5.1 Impact magnitude & immediacy** (immediacy refers to how soon an impact can be seen on the intended beneficiaries of the proposed research)	**5%**	**Good**	The proposed study made specific mention on the magnitude, reach and how soon the potential impact(s) are expected. There are multiple target group(s) / stakeholders that will clearly benefit from the study	**4**	**4.00**	
**5.2 Potential to generate tangilble outputs and impact** (as implied by the proposal and interpreted from the reviewers' point of view)	**5%**	**Good**	The proposal is explicitly clear on the expected skilled human resource, graduate students, publications, patents, policy paper and other measurable outcome/impact expected of the study. The research team is able to outlined the extent and type of otucomes/impact, as well as their target groups/stakeholders	**4**	**4.00**	
**5.3 Potential application(s) and follow up studies**	**5%**	**Good**	The proposal showed evidence on how knowledge garnered could be disseminated to, and applied by the target group(s). Follow up study is highly possible	**4**	**4.00**	
**5.4 Key outputs and highlights** (as declared in the proposal)<br><br>*Can also be adjusted by monetary return per paper, or IP*	**5%**	**Good**	The key outputs of the proposed research include graduate students, citation index publication in highly cited journals, new knowledge, IP's and probable policy paper drafts or product	**4**	**4.00**	
**5.5 Engagement Impact**	**2%**	**Average**	The proposal showed some degree of engagement impact with national community, authorities or industry, and international academic partners, apart from the local academia	**3**	**1.20**	
**6.0 Competency of the research team**						
**6.1 Track record (Qualification & experience** (in terms of grants and papers of researchers)	**2%**	**Good**	All team members in the team has published, held exisiting or former grants related to the research topics (or subtopics) proposed in the current study in the past 5 years. There are also evident collaboration (in terms of publication) between team members	**4**	**1.60**	

7.0 Research & Innovation Ecosystem (Data sharing plan and stakeholder engagement - international partner, industry, authorities, agencies and communities)						
**7.1 Stakeholder engagement & participation**	**2%**	**Average**	The proposal show some appreciable evidence of stakeholder involvement	**3**	**1.20**	
**7.2 Project sustainability** (Project is considered sustainable if it can sustain itself using other funds, or any other means to sustain itself beyond the proposed project period, without additional funding from the original sponsors)	**2%**	**Good**	Project is clearly sustainable beyond the proposed duration	**4**	**1.60**	
**7.3 Innovation and novelty**	**3%**	**Good**	The project has good elements of innovation and novelty, that could contribute significantly to its field of origin	**4**	**2.40**	
**7.4 Data sharing plan**	**2%**	**Below Average**	The proposal includes some description on how data derived from the research is to be shared within the research community only, but not with industrial stakeholders	**2**	**0.80**	
**8.0 Quality of proposal (Meticulous / spelling, grammatical and expression errors)**						
**8.1 Quality of proposal**	**5%**	**Average**	The proposal has some grammatical and other errors that won't otherwise compromise the aim and intent of the proposed study	**3**	**3.00**	
**JUMLAH MARKAH KESELURUHAN *(TOTAL SCORE)***					**70.19**	

**BELANJAWAN** *BUDGET*							
**Butiran belanjawan** *Budget details*	**Had** Limit (%)	**Syarat** *Conditions*	**Komen Panel** *Panel's Comment*	**Bajet yang dicadangkan oleh Panel *(RM)*** *Recommended Funding by Panel*			
				**Tahun 1** *Year 1*	**Tahun 2** *Year 2*	**Tahun 3** *Year 3*	**Jumlah Total**
**Vote 11000**	Tiada had *No limit*	Gaji dan upahan (Tidak terpakai untuk Geran Putra-IPM dan Putra-IPS) Salary and wages (Not applicable for Geran Putra-IPM and Putra-IPS)					
**Vote 21000**	Tiada had *No limit*	Perbelanjaan perjalanan dan pengangkutan *Travel expenses and transportation* Had untuk menghadiri seminar / konferensi; GPB & IPM : 10% GP-IPB : 20% GP-IPS : Tidak layak					
**Vote 24000**	Tiada had *No limit*	Sewaan *Rental*					
**Vote 26000**	Tiada had *No limit*	Bekalan bahan penyelidikan serta bekalan bahan mentah dan bahan-bahan untuk penyenggaraan dan pembaikan *termasuk vot 27000. *Research materials and materials for repair and maintenance*including vote 27000*		12,500	12,500		**25,000.00**
**Vote 28000**	Tiada had *No limit*	Penyelenggaraan dan pembaikan kecil *Maintenance and minor minor repair services*					
**Vote 29000**	Tiada had *No limit*	Perkhidmatan profesional, hospitaliti dan lain-lain Professional services, hospitality and other special services Had 10% untuk kos menghadiri kursus, program sangkutan sahaja.					
**Vote 35000**	**40%**	Peralatan dan aksesori (Sila rujuk garispanduan untuk keterangan lanjut) *Equipment and accessories (Please refer to guidelines for more information)*					
**JUMLAH PERUNTUKAN YANG DICADANGKAN (RM)** *Total recommended funding (RM)*				**12,500.00**	**12,500.00**	**0.00**	**25,000.00**
